# Preparation and Characterization of PLG Microparticles by the Multiple Emulsion Method for the Sustained Release of Proteins

**DOI:** 10.3390/mi13101761

**Published:** 2022-10-18

**Authors:** Arphaphat Yenying, Krissana Tangamatakul, Chayarop Supanchart, Thannaphat Jenvoraphot, Kiattikhun Manokruang, Patnarin Worajittiphon, Winita Punyodom, Donraporn Daranarong

**Affiliations:** 1 Department of Oral and Maxillofacial Surgery, Faculty of Dentistry, Chiang Mai University, Chiang Mai 50200, Thailand; 2 Bioplastic Production Laboratory for Medical Application, Faculty of Science, Chiang Mai University, Chiang Mai 50200, Thailand; 3 Department of Chemistry, Faculty of Science, Chiang Mai University, Chiang Mai 50200, Thailand; 4 Science and Technology Research Institute, Chiang Mai University, Chiang Mai 50200, Thailand

**Keywords:** microparticles, bovine serum albumin (BSA), encapsulation efficiency, poly(L-lactide-co-glycolide), multiple emulsion method

## Abstract

Rapid release and diminished stability are two of the limitations associated with the growth factors that are essentially used in dental applications. These growth factors are employed to enhance the quality and quantity of tissue or bone matter during regeneration. Therefore, drug delivery devices and systems have been developed to address these limitations. In this study, bovine serum albumin (BSA), as a representative growth factor, was successfully sustained by encapsulation with the medium-absorbable copolymer, poly(L-lactide-*co*-glycolide) (PLG) 70:30% mol, via the multiple emulsion method. Different PLG, PVA, and BSA concentrations were used to investigate their effects on the BSA encapsulation efficiency. The suitable ratios leading to a better characterization of microparticles and a higher encapsulation efficiency in producing encapsulated PLG microparticles were 8% (*w*/*v*) of PLG, 0.25% (*w*/*v*) of PVA, and 8% (*w*/*v*) of BSA. Furthermore, an in vitro release study revealed a bursting release of BSA from the encapsulated PLG microsphere in the early phase of development. Subsequently, a gradual release was observed over a period of eight weeks. Furthermore, to encapsulate LL-37, different proteins were used in conjunction with PLG under identical conditions with regard to the loading efficiency and morphology, thereby indicating high variations and poor reproducibility. In conclusion, the encapsulated PLG microparticles could effectively protect the protein during encapsulation and could facilitate sustainable protein release over a period of 60 days. Importantly, an optimal method must be employed in order to achieve a high degree of encapsulation efficiency for all of the protein or growth factors. Accordingly, the outcomes of this study will be useful in the manufacture of drug delivery devices that require medium-sustained release growth factors, particularly in dental treatments.

## 1. Introduction

Bone resorption after tooth removal can cause dental issues of an esthetic and/or functional nature. A number of different strategies have been explored in recent years for the treatment of bone resorption. One area of interest is the use of bioactive molecules, especially those related to growth factors (GFs), alone or in combination with other biomaterials, in order to address this problem [1]. GFs are known to stimulate cellular growth, proliferation, migration, and differentiation. There are various GFs that can essentially be used in modern biomedical applications in order to enhance the quality and quantity of the tissue during the regenerative process. A major limitation of the growth factors employed to induce tissue regeneration during wound healing is that they can be too rapidly released in the early phase of the bone repair process and can provide less stability in clinical applications. Many studies have focused on the growth factors that are released by more than 40% within the first day of the process [2,3]. While some studies have reported no bone formation during the first week after tooth extraction, it has been observed to begin on the eighth day [4]. This outcome corresponds with the findings of a study conducted by Farina et al., who reported that bone formation begins after the first week and can continue until the twelfth week after tooth removal [5]. Notably, the optimal degree of efficiency for the growth factors will not necessarily exist throughout the entire bone healing process. To improve the therapeutic efficiency of these growth factors, encapsulating them within carriers during the administration of drug delivery devices should be considered, and this could address the above-mentioned limitations. An encapsulation technique based on the water-in-oil-in-water (W/O/W) emulsion method or the multiple emulsion method would be best suited to encapsulate water-soluble drugs such as peptides, proteins, and vaccines [6]. The multiple emulsion method employs three important steps: (i) mixing proteins or drugs with the polymer solution to induce the primary emulsion or water-in-oil phase, (ii) adding the primary emulsion to an emulsion stabilizer, and (iii) evaporation or extraction of the organic solvent to initiate the hardening of particles [7,8]. Moreover, this method has been successfully applied for the entrapment of proteins and drugs within the microparticles that are usually produced by the biodegradable poly(L-lactide-*co*-glycolide) (PLG) polymer [7]. This polymer has been used for many years because it is known to be biodegradable and it can be degraded into nontoxic products in the human body [3].

Most studies related to addressing these issues have used biodegradable PLG, which is rich in glycolic acid or glycolide components (>50%mol). As a rule, a higher glycolide monomer content leads to a greater hydrophilic activity, resulting in faster degradation rates and a more significant initial burst [7,9]. PLG, which contains lactic/glycolic ratios of 10:90 and 50:50, could decrease the molecular weight much faster than PLG at ratios of 80:20, 90:10, and 10:90, and became totally fragmented after the sixth week. [10,11]. On the other hand, the poly (lactic acid) (PLA) polymer and the polycaprolactone (PCL) polymer were found to be associated with slower degradation rates at 2–5 years and 2–3 years, respectively [10]. Because the duration of the wound healing process carries on for about 4–6 months, the goal of establishing a polymer design for GFs or protein encapsulation would also be beneficial in terms of the degradation time. Moreover, it would also help to prevent a high number of proteins (>60%) from being released very quickly (24 h), which is one of the biggest problems associated with a controlled release system [12]. Therefore, a PLG containing a rich lactide (>60%mol) and medium glycolide (<40%mol) monomer ratio would be more suitable for this application than the PGA and PLA polymers [6]. To form the hardening particles, the primary emulsion is further emulsified with the addition of an emulsion stabilizer that is usually present in the aqueous phase and insoluble during the primary stage of emulsion. These would include poly(vinyl alcohol) (PVA) or poly(vinyl pyrrolidone) (PVP) [13]. The effectiveness of the encapsulation process is determined by encapsulation efficiency (EE), which refers to the successful entrapment of the agents within the particles [14]. There are various parameters that can influence the size of the particles and the degree of encapsulation efficiency. These factors would include protein or drug loading, polymer concentration, and emulsifier concentration [15].

Previous studies have reported that the PLG microparticles within the glycolic acid monomer ratio of >50%mol could effectively entrap proteins. Accordingly, the proteins were completely released from the PLG microparticles within a few days [1,16,17]. Moreover, it has been shown that the encapsulation of BSA is independent of the PLG molecular weight and also the PLG ratio [18]. Therefore, with different ratios or microstructures for PLG, the process parameters of the multiple emulsion method that could play a more important role on the encapsulation efficiency have to be evaluated. To avoid a relatively high release of proteins in a controlled way at the initial stage, in this study, PLG within a ratio of 70:30%mol (L-lactide:glycolide) was employed for the first time to investigate the encapsulated proteins via the multiple emulsion method for use in bone regenerative applications. In addition to characterization, the polymers, emulsion stabilizers, and protein concentrations were varied in order to investigate their effects on the protein encapsulation efficiency. The releasing profile of the proteins was also examined for a period of the wound healing process (>60 days). The bovine serum albumin (BSA) was used as a model protein because it is one of the most widely studied proteins, and it is inexpensive when compared with other growth factors [14]. Moreover, the reproducibility of the preparation method was determined through different protein encapsulation experiments prepared under similar conditions. We expect that the outcomes of this study will help researchers develop a prototype that can then be used in the manufacture of an effective drug delivery device used in conjunction with other potential growth factors in order to achieve sustained-release growth factors in dental treatments.

## 2. Material and Methods

### 2.1. Materials

PLG (Inherent Viscosity 0.3–1.0 dL/g) with a copolymer ratio of 70:30%mol (L-lactide:glycolide) was purchased from the Bioplastic Production Laboratory for Medical Applications, Faculty of Science, Chiang Mai University, Thailand. Poly(vinyl alcohol) (PVA, Mw:77000-88000) was purchased from Sigma Alrich. A bicinchoninic acid assay (BCA) protein assay kit Pierce^TM^ was purchased from Ward Medic (Bangkok, Thailand). Albumin obtained from bovine serum fraction V was purchased from S.M. Chemical supplies (Bangkok, Thailand). Synthesized LL-37 (LLGDFFRKSKEKIGKEFKRIVQRIKDFLRNLVPRTES) was purchased from Chempeptide Limited (Shanghai, China). LL-37 ELISA kits were purchased from Hycult^®^ Biotech, Inc. (Uden, The Netherlands). Dichloromethane (DCM) solvents (AR grade, LabScan, Bangkok, Thailand) were used as they were received. 

### 2.2. Microparticle Preparation

#### 2.2.1. Effect of PLG Concentration 

Microparticles were prepared by employing the multiple emulsion method by following the process described in previous experiments, with slight modifications [14]. Various concentrations of the PLG copolymer (4.0−12.0% (*w*/*v*)) were dissolved in 10 mL of DCM. Accordingly, 40 mg of BSA was dissolved in 10 mL of distilled water and mixed into each PLG solution. The mixtures were then stirred with a magnetic bar for 15 min at a speed of 1200 rpm. This organic phase solution was gradually dropped into a dissolution of 2.0% (*w*/*v*) PVA, which was used as an emulsion stabilizer. Subsequently, it was set in distilled water in order to generate a secondary (W/O/W) emulsion. The secondary emulsion was then continuously stirred for 24 h at 40 °C in order to evaporate the organic solvent and harden the particles. The particles that were obtained by filtration were then washed with distilled water and vacuum-dried for 24 h. 

#### 2.2.2. Effect of PVA Concentration

To investigate the influence of the PVA concentration, the amounts of BSA and polymer concentrations were held constant. Microparticles were then prepared by employing the same procedure, except for the use of different PVA concentrations (0.10–2.50% (*w*/*v*)) and by dissolving 8% (*w*/*v*) PLG in DCM.

#### 2.2.3. Effect of BSA Concentration

To investigate the influence of BSA concentration, the amounts of polymer concentration and PVA were held constant. Microparticles were then prepared by employing the same procedure, except for the use of different BSA concentrations within a range of 0–8% (*w*/*v*) and by dissolving 0.25% (*w*/*v*) PVA in distilled water. 

### 2.3. Microparticle Characterization

#### 2.3.1. Determination of Encapsulation Efficiency of Microparticles

The encapsulation efficiency was calculated indirectly by the amount of free BSA present in the clear supernatant after filtration of the microparticle suspension. Supernatants were collected in order to measure the non-encapsulated BSA via spectrophotometry (UV 3600 Shimadzu Spectrophotometer) using a BCA protein assay kit (Pierce, Rockford, IL, USA) at 540 nm [19]. The encapsulation efficiency (%) of BSA into the microparticles was determined as follows:

Encapsulation Efficiency (%) = (A/B) × 100

where A = BSA protein entrapped in microparticle (mg), and B = initial loaded amount of BSA protein (mg).

#### 2.3.2. Determination of Particle Morphology and Chemical Structure

Scanning electron microscopy (SEM) is an electron optical imaging technique that yields both topographic images and elemental information. It is used in conjunction with energy-dispersive X-ray fluorescence (EDXRF). Therefore, in this work, SEM (JSM 5910 LV Scanning Electron Microscope, JEOL Ltd., Tokyo, Japan) attached with EDXRF (INCA Energy Software, Oxford Instruments, Abingdon, UK) was used to observe the morphology and chemical composition of the BSA encapsulated in the PLG particles, along with their particle diameters. Gold sputtering was used to make the encapsulated particles conductive for the SEM-EDXRF investigations.

In order to obtain additional structural information, FTIR in an attenuated (ATR) mode was used to confirm the presence of the BSA encapsulated in the PLG particles.

### 2.4. In Vitro BSA-Released Study

Various amounts of BSA were loaded into the PLG microparticles by following the method previously employed in the experiment described in Section 2.2.3. These amounts were accurately pre-weighed for in vitro hydrolytic degradation studies. The samples were then dispersed in 10 mL of phosphate buffer solution (PBS) containing 0.1% NaN_3_ at a pH of 7.4 for preservation in 15 mL microcentrifuge tubes. The suspensions were then incubated at 37 °C under shaking conditions for eight weeks. At designated time intervals throughout the 8-week period, the supernatants (10 mL) were collected and separated by centrifugation (500× *g* for 10 min at 4 °C) to determine the amount of BSA released. Accordingly, 10 mL of fresh PBS, along with 0.1% NaN_3_, were added to the solution. The amount of released BSA proteins per time point was determined via spectrophotometry (UV 3600 Shimadzu Spectrophotometer) using a BCA protein assay kit (Pierce, Rockford, IL, USA) at 540 nm, and was expressed as the cumulative percentage of BSA protein release.

### 2.5. Reproducible of Protein Encapsulation 

The antimicrobial peptide, LL-37, was chosen as a representative protein for bone regeneration in order to evaluate the reproducibility of the microparticle preparation method under similar conditions. Accordingly, 40 mg of LL-37 was dissolved in 10 mL of distilled water and mixed into the PLG solution. The LL-37-PLG microparticles were then also prepared following the same procedure. Afterwards, the microparticles were characterized in terms of the loading efficiency and morphology. Human LL-37 ELISA kit (Hycult^®^ Biotech) was used to determine the LL-37 in the supernatants.

### 2.6. Statistical Analysis

All of the data were statistically evaluated using two-way ANOVA analysis and Bonferroni post hoc test with statistical significance levels established at *p* < 0.05.

## 3. Results and Discussion

### 3.1. Microparticle Preparation and Characterization

#### 3.1.1. Effect of PLG Concentration

**Figure 1:**
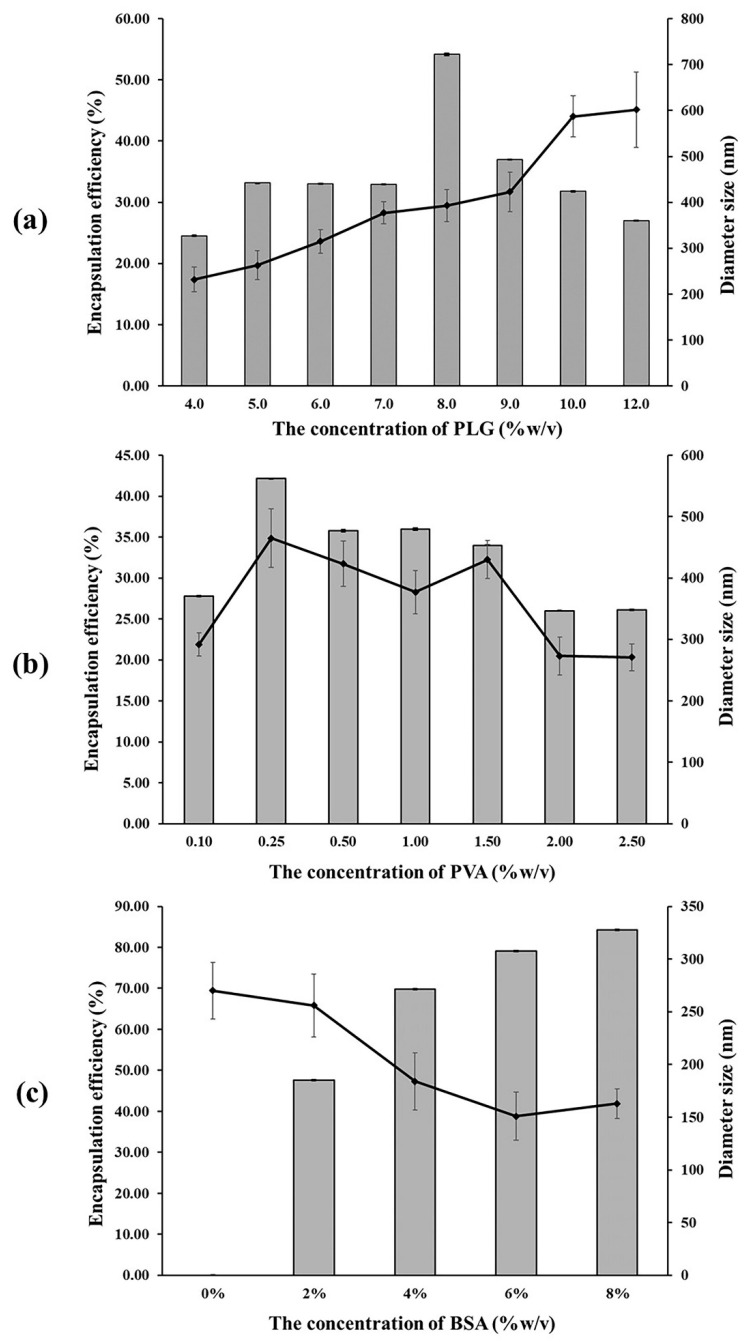
Encapsulation efficiency (%) (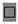) and diameter size (nm) (**-**◆**-**) of the BSA encapsulated in the PLG microparticles with various concentrations of (**a**) PLG, (**b**) PVA, and (**c**) BSA (%*w*/*v*).

Encapsulated poly(L-lactide-*co*-glycolide) (PLG) polymer microparticles were prepared by employing the multiple emulsion method. The average diameter established by the ImageJ program showed diameters within a range of 239–602 nm. This was indicative of a larger size within the higher ratio of the polymer concentration group, as is shown in Figure 1a. The SEM images show that the microparticles were characterized by a smooth surface and are spherical in shape. It was determined that the microparticles tended to have a more spherical shape within a higher ratio of the polymer concentration group. 

Encapsulation efficiency (EE) is defined as the ratio of proteins encapsulated in the microparticle. It is dependent on the polymer concentration, as has been presented in Figure 1a. The results indicate a discrepancy in the encapsulation efficiency between different PLG concentrations using the multiple emulsion method. Encapsulation efficiency tends to be increased when the ratio of the polymer concentration is higher. This result is in accordance with the outcomes of a study conducted by Rafati et al., who determined that the degree of BSA encapsulation efficiency increased when the PLG concentration increased from 1% to 6% via the multiple emulsion method [20]. Although the highest degree of encapsulation efficiency (54.16% ± 0.15%) was observed in the 8% (*w*/*v*) PLG group, it was found to have decreased in the 9% (*w*/*v*) PLG group. The degree of encapsulation efficiency of the 8% (*w*/*v*) PLG group was greater than 50%, which was required for this study. Therefore, we used 8% (*w*/*v*) PLG to investigate the influence of the PVA concentration in our experiments. The higher ratio of the polymer concentration, which can make the polymer more viscous, allowed the microparticles to be hardened more slowly via the multiple emulsion method, resulting in the formation of microparticles with a better shape and surface, as well as microparticles that are less porous, thus allowing for greater protein entrapment [21,22]. Moreover, raising the polymer concentration would generate a thicker polymer film around the protein, which would then facilitate the protein to be completely entrapped in the polymer and could prevent drug loss in the external aqueous phase, resulting in an increasing encapsulation efficiency [23,24]. 

#### 3.1.2. Effect of PVA Concentration

In the multiple emulsion method, the organic solvent should be removed completely from the hardening particle during the microencapsulated process. This study used PVA as an emulsion stabilizer to extract the organic solvent particles during the hardening particle process [13]. The average diameter was within a range of 271–465 nm, for which the largest particle was found in the 0.25% (*w*/*v*) PVA group, and tended to decrease at a higher ratio for the PVA concentration, as is shown in Figure 1b. The SEM image revealed that the smoothest surface of the microparticles was observed in the 0.25% (*w*/*v*) PVA group. However, the surface of microparticles did present an irregular shape and a rough surface in the groups that used a PVA concentration of more than 0.25% (*w*/*v*). Figure 1b shows the encapsulation efficiency at different PVA concentrations. Accordingly, it can be seen that the 0.25% (*w*/*v*) PVA group achieved encapsulation efficiency at 42.18% ± 0.01%. Nevertheless, the encapsulation efficiency tended to decrease when the PVA concentration was more than 0.25% (*w*/*v*). This result corresponds to that of a study conducted by Conti et al., which evaluated three parameters in the preparation of indomethacin loaded PLG microspheres. They reported that the degree of encapsulation efficiency was high when a low level of PVA concentration was used in the system [15]. This would suggest that the protein can be soluble in the external phase and then easily leached into the external aqueous phase of the PVA. This outcome resulted in extensive protein loss during the encapsulation process, as well as a low level of encapsulation efficiency [13]. On the other hand, this study found that the degree of encapsulation efficiency was low in the PVA concentrations that were lower than 0.25% (*w*/*v*). This could have occurred as a consequence of the level of PVA concentration being too low to extract the entire organic solvent, which would then result in a low degree of encapsulation efficiency.

#### 3.1.3. Effect of BSA Concentration

**Figure 2:**
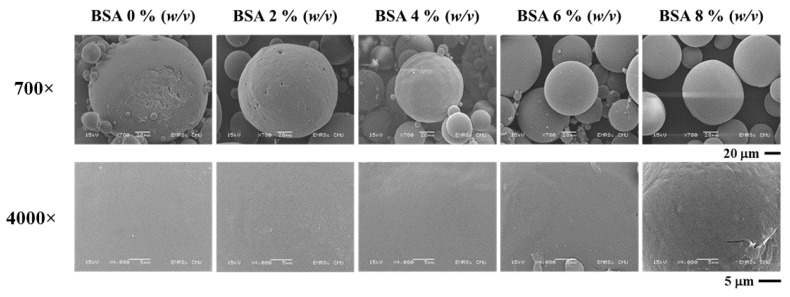
SEM image of BSA encapsulated in PLG microparticles with various concentrations of BSA.

To investigate the optimal degree of BSA concentration in the encapsulation efficiency experiment, the average diameter was shown to be within a range of 151–270 nm. The results indicate that a value larger than 0% (*w*/*v*) tended to decrease in size at higher concentrations of BSA. The SEM image (Figure 2) revealed the smoothest surface of the microparticle in the 0% BSA (*w*/*v*) group. Furthermore, a rough surface and irregular shape were observed at higher concentrations of BSA. The results presented in Figure 1c indicate that the degree of encapsulation efficiency increased according to the BSA concentration. The highest degree of encapsulation efficiency was observed at 84.26% ± 0.02% in the 8% (*w*/*v*) group. Thus, it has been hypothesized that BSA concentrations and the degree of encapsulation efficiency were determined to be direct variations. This could have occurred because a higher protein concentration contributed to a higher osmotic gradient. For this reason, the diffusion rate of the water from the external phase to the primary emulsion was higher than the diffusion rate of water from the primary emulsion to the external phase, which then resulted in a higher degree of encapsulation efficiency [24].

### 3.2. Microparticle Characterization

The complete BSA encapsulation in the PLG microparticles was confirmed by SEM images. Moreover, the EDXRF elemental analysis data confirmed the presence of carbon (C) and nitrogen (N), as shown in Table 1. Therefore, BSA encapsulation in the PLG microparticles was deemed to be successful. Furthermore, the Fourier transform infrared spectroscopy (FTIR) spectra of the PLG microparticles at various concentrations of BSA encapsulation were obtained using a Nicolet™ iS™ 5 FTIR Spectrometer (Thermo Fisher Scientific Inc., USA). The attenuated total reflectance (ATR) mode was used to record spectra with 32 scans over a range of 400–4000 cm^−1^ at a resolution of 2 cm^−1^, to produce the vibrational assignment data shown in Table 1.

Figure 1 shows all of the FTIR spectra of PLG microparticles at various concentrations of BSA encapsulation that indicate the presence of carbonyl groups (CH, CH_2_, and CH_3_) within a range of 2981–3010 cm^−1^. Other major peaks observed for the encapsulated microparticles were C-O stretch (1737–1753 cm^−1^), C-O stretch (1102–1134 cm^−1^), and O-H stretch (3750–3764 cm^−1^). There were no FTIR spectra that indicated the peak of the −NH groups in all of the PLG microparticles at various concentrations of BSA encapsulation. It was thus suggested that there may be a coordinated interaction between the −OH groups in the polymer and the −NH groups in the BSA, which would likely play an important role in the formation of PLG microparticles.

### 3.3. BSA-Released Behavior Study

**Figure 3:**
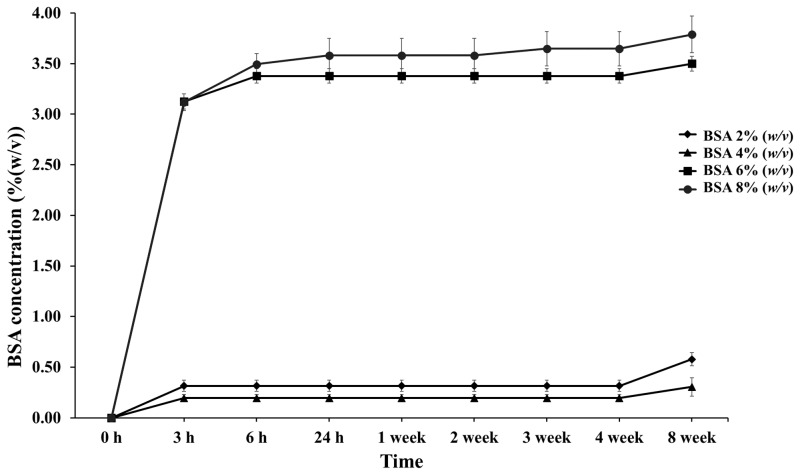
Percentage of cumulative release profiles of PLG microparticles with various concentrations of BSA encapsulation at designated time intervals throughout the 8-week period.

The in vitro release of BSA was investigated from PLG microparticles prepared with different BSA concentrations at constant PVA and PLG concentrations. The values were measured for 30 days in a physiological buffer solution (PBS) containing 0.1% NaN_3_ at a pH of 7.4 for preservation at 37.0 °C ± 0.5 °C. The BSA release from the PLG microparticles is presented in Figure 3. The initial release occurred as a burst within 3 h in every group and the release was thereafter sustained. Burst release values of 0.32%, 0.2%, 3.13%, and 3.12% were recorded in 2%, 4%, 6%, and 8% (*w*/*v*) BSA, respectively, over the course of 3 h. Accordingly, values of 1.12, 0.7, 10.96, and 10.92 milligrams for the protein concentration were then recorded. This graph indicates a higher burst release when the concentration of BSA increased. It also shows a gradual release in the 6% and 8% (*w*/*v*) BSA group between 3 and 6 h. This outcome is in accordance with the results of a study conducted by George C. et al., who investigated the release of BSA and CM-BSA. They showed that protein BSA and CM-BSA presented a burst release value of up to 10% during the first day. It was then slowly and continuously released thereafter [25]. Moreover, a lag phase was observed in every group, but the 8% (*w*/*v*) BSA group showed a release of 0.07% again at the end of the third week. A second release was observed in the 8% (*w*/*v*) BSA group, which corresponds with the outcomes of a number of previous studies that reported a biphasic release of PLG polymer encapsulation. The biphasic release is indicative of the releasing behavior of the protein encapsulated in the PLG polymer composed at the initial phase (initial burst) and the second phase of the protein release process [26].

The initial phase or initial burst of protein release resulted in the presence of proteins on the surface of the microparticles or observed at the point of surface diffusion. This was caused by the penetration of water into the polymer matrix, resulting in protein solubility and a subsequent release into the media. However, the BSA releasing process occurred via both surface diffusion and polymer degradation. The degradation of the PLG polymer was composed of a combination process of bulk erosion and the surface erosion of the polymer, which were degraded by hydrolysis through its ester linkages. Hydrolysis is the process wherein water penetrates to the matrix, cleaving the polymer into specific soluble products, namely lactic acid and glycolic acid [27]. This creates a passage for the drug to be released by diffusion and erosion until complete polymer solubilization. The degradation of the polymer occurs after the initial burst process appears, as this process can require time.

The cumulative release of BSA (Figure 3) in the 6% and 8% (*w*/*v*) BSA groups was higher than in the others, which increased to 3.38% and 3.65% over three weeks. BSA continued to be released until the fourth week of the experiment. It is believed that with increasing the concentrations of proteins and/or drugs, the dissolved protein in the polymer solution exhibited a greater tendency to migrate to the surface or near the surface of the PLG microparticles during the encapsulation process. Therefore, the exposure and diffusion of BSA encapsulated in the PLG microparticles to the buffer solution tended to increase during the third and fourth weeks, leading to a faster release rate. Moreover, the sustained release lasted for eight weeks. It was determined that the BSA release profile was sustained by encapsulation in the PLG polymer via the multiple emulsion method. Thus, PLG within a ratio of 70:30%mol (L-lactide:glycolide) microparticles could control the release of BSA to assist in the wound healing process, and specifically in the tissue and bone regeneration processes that begin after the first week and continue up until the twelfth week after tooth extraction [4,5]. We contend that these designated conditions can be used with other growth factors related to bone healing and bone regeneration enhancement, such as vascular endothelial growth factors (VEGFs), platelet derived growth factor (PDGF), bone morphogenic proteins (BMPs), and antimicrobial peptides [28].

### 3.4. Reproducible of Protein Encapsulation 

**Figure 4:**
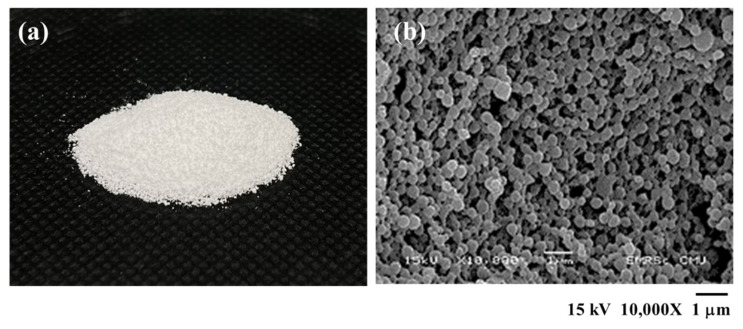
(**a**) Photograph and (**b**) SEM image of LL-37-encapsulated in PLG microparticles with 8% (*w*/*v*) of PLG, 0.25% (*w*/*v*) of PVA, and 4% (*w*/*v*) of LL-37.

The reproducibility of the preparation method was determined through different protein encapsulation experiments prepared under similar conditions. The antimicrobial peptide, LL-37, was evaluated as a representative protein for bone regeneration in order to evaluate the reproducibility of the microparticles. LL-37 is a human antimicrobial peptide in the cathelicidin family and it is present in the innate immune system. LL-37 consists of 37 amino acids at the C-terminal of the human cationic antimicrobial peptide (hCAP18) which is produced by neutrophils, epithelial cells, gingival epithelial cells, the respiratory tract, and the gastrointestinal tract, all of which can serve as physical barriers against infection. Many studies have reported that LL-37 can promote MSCs proliferation, osteogenic differentiation, and bone regeneration [29,30,31,32]. In this study, the LL-37 proteins encapsulating PLG microparticles were prepared under the best conditions obtained from BSA evaluation, including 8% (*w*/*v*) of PLG, 0.25% (*w*/*v*) of PVA, and 4% (*w*/*v*) of LL-37. The outcomes indicated that the preparation method could result in high variations and poor reproducibility. The degree of percentage encapsulation efficiency and the average diameters did not produce similar results when compared with the BSA protein. The average diameter of the LL-37 protein encapsulated PLG microparticles was within a range of 20–56 nm, which was smaller than that of the BSA protein by 92%. These microparticles are suitable for oral delivery, mucosal adhesion, or inside scaffold use, as well as for bone regeneration. The nanoscale dimension of the carrier offers enhanced versatility when compared with particles of a larger size [1]. The SEM image (Figure 4) revealed a rough surface and irregular shape. Moreover, the degree of percentage encapsulation efficiency of the LL-37 encapsulated PLG particles was 6.61 ± 0.92, which was lower than that of the BSA encapsulated in the PLG microparticles. As a general rule, encapsulation efficiency increases along with the size of the particles [9]. It can be assumed that the parameters or conditions that are used in BSA protein encapsulation are not necessarily suitable for LL-37 or other proteins. This could be explained by the limitations of the multiple emulsion method. Notably, various parameters would likely be more appropriate for the specific properties of certain microparticles. These include the polymer concentration, speed of the stirrer, and the time and volume ratio. Moreover, the structure of the encapsulated protein molecules can be affected, while the denaturation processes and a loss of biological activity can appear when they interact with an organic solvent such as DCM [33]. Thus, the optimization of this method must be established in order to achieve a high degree of encapsulation efficiency for all proteins and/or GFs as a primary goal.

## 4. Conclusions

The encapsulation of microparticles achieved in the medium absorbable copolymer, poly(L-lactide-*co*-glycolide) (PLG) with a 70:30% mol ratio, has been based on the water-in-oil-in-water method or the multiple emulsion method. This process has been used extensively and effectively. The 70:30 mol% of PLG was developed to improve the therapeutic efficiency of the specific growth factors used in modern biomedical applications in order to enhance the quality and quantity of tissue matter during regeneration in dental treatments. This study identified the appropriate concentrations of the polymer, the emulsion stabilizer, and the BSA’s impact on microparticle characterization including the size of particles, their morphology, and their impact on encapsulation efficiency. The results of this study indicate that the most suitable concentrations, including better characterization of the microparticles and a higher degree of encapsulation efficiency, for the production of encapsulated PLG microparticles, were 8% (*w*/*v*) of PLG, 0.25% (*w*/*v*) of PVA, and 8% (*w*/*v*) of BSA. Furthermore, this PLG copolymer medium can be used for the manufacture of drug delivery devices in conjunction with other potential growth factors in order to achieve a higher degree of therapeutic efficiency and for the sustainable release over a period of 60 days. Moreover, the same conditions associated with the encapsulation of PLG in the BSA protein may not be reproducible and suitable for the preparation of different proteins or GFs. In this study, the LL-37 protein encapsulated within the PLG microparticles was within the range of the other nanoparticles and exhibited a lower degree of % encapsulation efficiency. This was due to the limits of the multiple emulsion method. Accordingly, the optimization of the method must be considered in order to achieve a high degree of encapsulation efficiency for every protein or GF. Importantly, we expect that this study will be particularly useful in the development of medical pharmaceuticals, especially with regard to those employed in dental treatments.

## Figures and Tables

**Table 1:** EDXRF elemental analysis and data vibrational assignments of the PLG microparticles with various concentrations of BSA encapsulation.

BSA (%*w*/*v*)	Atomic (%)		Wavenumber (cm^−1^)			
	C	N	C-H Stretching	C=O Stretching	C-O Stretch	O-H Stretching
**0**	100.00	-	3010	1737	1134	3764
**2**	63.58	36.42	3009	1744	1121	3760
**4**	62.77	37.23	2986	1748	1116	3755
**6**	66.56	33.44	2982	1750	1105	3751
**8**	65.75	34.25	2982	1753	1102	3750
